# Unveiling the Nexus: Sulphur Dioxide Exposure, Proximity to Mining, and Respiratory Illnesses in Kankoyo: A Mixed-Methods Investigation

**DOI:** 10.3390/ijerph21070850

**Published:** 2024-06-28

**Authors:** Sipiwe Chihana, Jameson Mbale, Nchimunya Chaamwe

**Affiliations:** School of ICT, Copperbelt University Jambo Drive, Riverside, Kitwe P.O. Box 21692, Zambia; jameson.mbale@cbu.ac.zm (J.M.); chimz@cbu.ac.zm (N.C.)

**Keywords:** sulphur dioxide exposure, mining and health impact, respiratory illnesses, community health monitoring, mixed-methods research, air pollution

## Abstract

The emission of sulphur dioxide (SO${\;\;}_{2}$) from mining activities presents significant health hazards, particularly to communities near industrial zones. This mixed-methods study investigates the nexus between (SO${\;\;}_{2}$) exposure and respiratory health in Kankoyo Township, Zambia. Employing community engagement, expert interviews, spatial analysis, and a retrospective examination of 15 years of health and (SO${\;\;}_{2}$) data, the research identified a troubling correlation between (SO${\;\;}_{2}$) exposure and adverse respiratory health effects among the local population. Expert interviews highlighted that respiratory issues constituted approximately 75% of health complications, with a notable reduction in asthma cases following the installation of a monitoring station and upgrades to smelter operations. Spatial analysis demonstrated that (SO${\;\;}_{2}$) levels in Kankoyo exceeded the Zambian Environmental Management Agency (ZEMA) limits by 1713% identifying it as a significant pollution hotspot. Additionally, wind profile analysis indicated frequent low-speed winds from the east-northeast (ENE), contributing to pollutant accumulation. Based on these insights, the study recommends implementing real-time pollution data sharing, affordable air quality sensors, addressing medication shortages, establishing specialized respiratory clinics, launching IT-driven awareness campaigns, and further research into additional pollutants and confounding factors.

## 1. Introduction

The World Health Organization [1,2] has recognized air pollution as a significant worldwide environmental issue that presents substantial hazards to human health. The risk is especially significant for communities living near industrial sites, such as mining operations. In the Copperbelt Province of Zambia, areas such as Kankoyo in Mufulira are confronted with the ramifications of atmospheric contamination, particularly the release of sulphur dioxide (SO${\;\;}_{2}$). Mwaanga et al. [3] identify SO${\;\;}_{2}$ as the most critical air pollutant that continues to be emitted from mining companies in Zambia. The main source of this SO${\;\;}_{2}$ pollution is the roasting and refining of copper-bearing sulphide ores. The initial stage in most processes involves roasting or smelting the ore in the air, which oxidizes some of the copper and produces SO${\;\;}_{2}$. The authors further note that the increase in SO${\;\;}_{2}$ emissions has always been observed to increase at the same rate as copper production, primarily due to old, inefficient smelting processes [3]. Sulphur dioxide is a colourless gas with a pungent odour. When inhaled, it can cause a range of respiratory problems, including the irritation of the nose, throat, and airways, leading to symptoms like coughing, wheezing, and shortness of breath [4,5]. Studies have also shown that even short-term exposure to SO${\;\;}_{2}$ can exacerbate respiratory illnesses, especially in vulnerable populations such as individuals with asthma or the elderly [6]. Additionally, several global studies, such as those by [7,8], consistently demonstrate a strong relationship between ambient air contamination and negative impacts on lung health. Notwithstanding the prevailing global consensus, there is a notable paucity of local awareness about sulphur dioxide exposure and its precise ramifications on respiratory well-being in Zambia’s Copperbelt Province. The lack of comprehension impedes the precise evaluation of the true health consequences for communities living near mining operations [9]. As a result, it is critical to acknowledge and address this knowledge deficiency to alleviate the respiratory health hazards faced by these populations adequately. Kankoyo exemplifies this knowledge deficit, as its inhabitants frequently report respiratory ailments and other health complications linked to air pollution. Although several studies, such as the ones conducted by Muma, Mwaanga, and Nachalwe [3,10,11] and other researchers, have suggested a potential association between air pollution resulting from mining activities and health consequences in Zambia, there is a paucity of comprehensive research, especially research investigating the impact of SO${\;\;}_{2}$ exposure on respiratory health. This gap hinders our ability to comprehensively comprehend the effects of SO${\;\;}_{2}$ on population health in Kankoyo and comparable regions. Researchers in the past have painted a worrying picture. For example, Munshifwa [12] found that people living in Kankoyo had linked several health problems, including death, to air pollution in the area. The authors of the preliminary study on air pollution from mining operations in Zambia [3] also pointed out that pulmonary tuberculosis (PTB) and other breathing problems may be linked to mine air pollution in places like Mufulira. A television program titled “The Digest”, aired on 30 July 2022, by Diamond TV 2022 [13], a privately owned television station, highlighted the continued battle for clean air in Kankoyo, despite ongoing attempts to reduce pollution. The video emphasized the persistent resilience of residents in dealing with health problems like asthma caused by SO${\;\;}_{2}$ emissions, emphasizing the pressing necessity for more profound comprehension and efficient remedies.

### 1.1. Problem Statement

Numerous studies have indicated a potential correlation between air pollution and respiratory health issues in Zambia. However, there exists a shortage of comprehensive research specifically investigating the link between exposure to sulphur dioxide (SO${\;\;}_{2}$) and respiratory ailments within mining regions in Zambia. The shortage of extensive study in this domain is a substantial barrier to adequately tackling the health challenges encountered by communities, specifically those dwelling in close localities to the mine, such as Kankoyo. Hence, it is crucial to expeditiously conduct an extensive investigation to fully comprehend the exact impacts of SO${\;\;}_{2}$ exposure on respiratory well-being among these demographics.

### 1.2. Research Question

To fill the existing research gap, the primary objective of this study was to investigate the relationship between sulphur dioxide (SO${\;\;}_{2}$) exposure and the prevalence of respiratory illnesses in Kankoyo while considering the proximity to the mining site. To gain a full understanding of this link, we defined the primary research question and its corresponding sub-questions as follows:

#### Main Research Question

How does sulphur dioxide (SO${\;\;}_{2}$) exposure contribute to the occurrence of respiratory illnesses in Kankoyo, considering its proximity to the mining site?

Sub-questions 

(a) What factors influence the levels of sulphur dioxide (SO${\;\;}_{2}$) exposure in Kankoyo, particularly concerning the proximity to the mining site?
(b) What correlations exist between variations in sulphur dioxide (SO${\;\;}_{2}$) exposure levels and the incidence of respiratory symptoms among residents of Kankoyo?
(c) What is the level of awareness among Kankoyo residents regarding the potential health risks associated with SO${\;\;}_{2}$ exposure?
(d) What trends or patterns are observed in the prevalence of respiratory illnesses in Kankoyo over time and corresponding levels of SO${\;\;}_{2}$ exposure?

### 1.3. Area of Study

**Figure 1:**
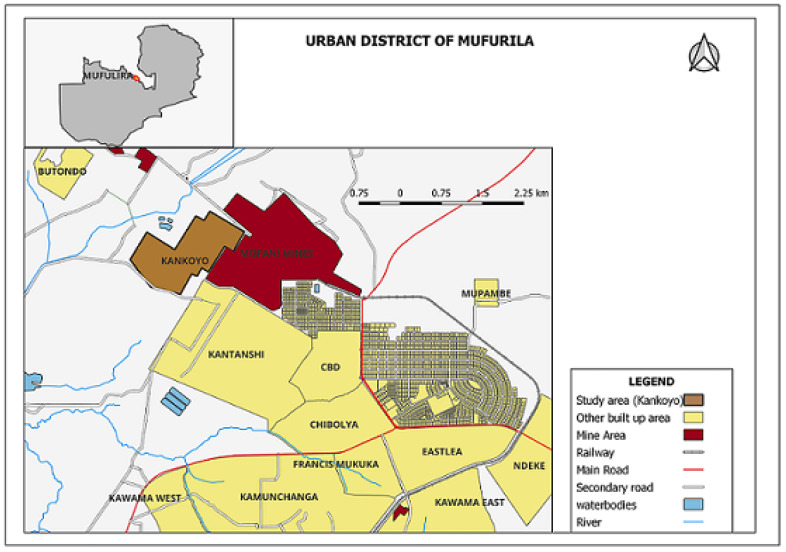
Study Area: Mufulira Kankoyo.

Located on the western outskirts of Zambia’s Mufulira District, Kankoyo bustles with a densely populated community of around 44,004 [14] inhabitants. Established in the 1930s to accommodate mine workers, this historic township lies in the direct path of the prevailing wind from Mopani Copper Mine Smelter. Situated at an elevation of 1288 m, its terrain gently rises and falls between 1250 and 1400 m, with a network of streams draining towards the Kafue River (Figure 1). Despite having affordable housing, Kankoyo’s economic environment heavily favours informal employment [10].

## 2. Literature Review

### 2.1. Mining and Air Pollution in Zambia

The mining industry worldwide is crucial for providing essential resources needed in construction, technology, and energy production [15]. However, this industry also raises environmental concerns. Copper production, a complex multi-stage process [16,17]), releases significant amounts of sulphur dioxide (SO${\;\;}_{2}$) that can harm vegetation and buildings when combined with rain [16], further affecting human health.

Zambia’s economy has been heavily reliant on its abundant copper reserves and thriving extractive industry for nearly a century [18,19]. While the recent surge in mining activity brings hope for development, the Copperbelt region grapples with a legacy of environmental mismanagement [3,10]. This long history of mining has resulted in significant air pollution, posing various health risks to residents through inhalation, contaminated food and water sources, and direct contact with polluted soil [20,21].

Sulphur dioxide (SO${\;\;}_{2}$) emissions from mining operations pose a major environmental and health threat in Zambia [3,10]. Despite being a prevalent issue since the early 20th century, the full extent and impact of SO${\;\;}_{2}$ pollution remain inadequately understood [3]. Copper smelters release not only uncaptured SO${\;\;}_{2}$ but also substantial particulate matter, particularly from tailing dams. Prevailing wind patterns further disperse these pollutants, exposing nearby communities to potentially harmful levels. SO${\;\;}_{2}$ can severely impact respiratory health, contributing to various respiratory illnesses like bronchitis and tuberculosis [3].

The heightened vulnerability of populations residing near mining sites is a critical concern. Studies like the Copperbelt Environmental Project [20] revealed significantly higher rates of respiratory infections among Copperbelt children than the national average. This increase, then particularly pronounced in Mufulira and Kitwe, was linked to elevated SO${\;\;}_{2}$ levels [20]. Similarly, a recent study in the Nkana West and Kankoyo townships reported that residents identified respiratory tract infections (RTIs) as their primary health concern, attributing it to mining activity [11].

The broader literature on mining and air pollution presents a troubling picture. Studies specifically conducted in Kankoyo, the community at the centre of this research, further emphasized this issue. These localized findings underscored the significant health concerns associated with mining-related air pollution. Thus, it was imperative to examine related research efforts to understand and mitigate the impacts of such environmental challenges.

### 2.2. Previous Findings in Kankoyo

Studies specifically focused on the Kankoyo community have raised significant concerns about health impacts associated with ambient air pollution. Muma [10] determined that the main causes were sulphur dioxide (SO${\;\;}_{2}$) and acid fumes from the smelting and sulphuric acid production plants at the Mopani Copper Mine. These industrial operations include several variables that affect SO${\;\;}_{2}$ emissions, such as ore quality fluctuations, smelting efficiency, and the efficacy of pollution control systems at the smelter. Sulphuric acid production is situated right across from Kankoyo Township, and if improperly contained, could leak SO${\;\;}_{2}$, which adds to the pollution burden. In addition to these specific sources, Muma [10] also highlighted the contribution of dust from bare, un-vegetated land to the overall air pollution burden in Kankoyo. While not directly related to SO${\;\;}_{2}$ emissions, this factor interacts with industrial pollution to create a complex air quality profile in the township. Furthermore, several other studies have documented the broader environmental and infrastructural impacts of mining-related air pollution in Kankoyo, highlighting the presence of various pollutants, including particulate matter (PM) and heavy metals. Table 1 summarizes these studies’ key findings and limitations, providing a broader context for understanding the environmental health challenges the Kankoyo community faces.

The research we examined indicated that mining-related air pollution in Zambia’s Copperbelt region is a significant issue. Numerous studies have identified several pollutants, such as SO${\;\;}_{2}$, dust, and heavy metals, and their potential adverse effects on human health, plant life, animal welfare, and infrastructure. Our understanding of the exact effects of SO${\;\;}_{2}$ on human respiration, especially in populations near mines, is inadequate. Research undertaken in regions like Kankoyo has indicated a potential correlation between respiratory problems and SO${\;\;}_{2}$. However, these studies did not comprehensively investigate the extent of SO${\;\;}_{2}$ exposure among individuals or the enduring health consequences. Furthermore, the majority of studies conducted in Kankoyo concentrated on environmental management and the economic impact of pollution rather than delving into its direct effects on human health. The lack of understanding in this area poses challenges in devising strategies to safeguard the well-being of communities residing close to mining sites. Hence, more investigation is needed to comprehend the extent of SO${\;\;}_{2}$ exposure and its impact on respiratory health within these populations.

## 3. Materials and Methods

**Figure 2:**
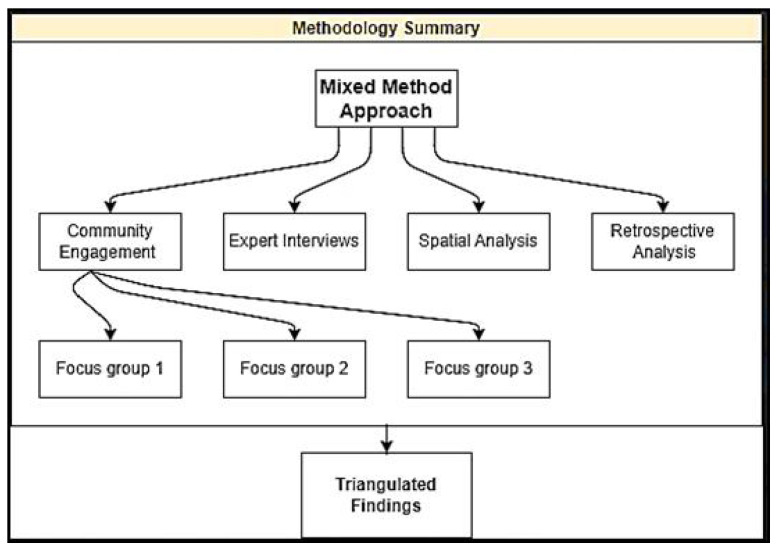
Research Study Blueprint Summary.

To uncover the nexus between sulphur dioxide (SO${\;\;}_{2}$) exposure and respiratory health in Kankoyo Mufulira, we devised a comprehensive research strategy. Our approach integrated various methods to effectively gather information. Initially, focus groups with residents of Kankoyo were conducted to collect insights on their experiences with air quality, concerns regarding mining operations, and potential exposure to SO${\;\;}_{2}$. The narratives from these groups provided invaluable perspectives that enriched our methodological analysis. Additionally, we carried out a series of interviews with environmental and public health experts to gain scientific insights into the effects of mining on air pollution within the Kankoyo Mufulira region. The expertise of these professionals significantly enhanced our understanding of the intricate dynamics involved. Furthermore, we utilized spatial analysis to identify areas with elevated pollution levels and to examine the wind profile of the study area. This method enabled us to establish correlations between environmental variables, such as wind direction and speed, and regions particularly vulnerable to pollution. Incorporating this geographical perspective augmented the depth of our analytical framework. We also performed a retrospective analysis of long-term health data sourced from the local healthcare facility. This analysis aimed to identify patterns and associations between historical SO${\;\;}_{2}$ exposure levels and health outcomes within the community. The primary objective of our study was to investigate the ramifications of SO${\;\;}_{2}$ in Kankoyo Mufulira and to develop evidence-based interventions for promoting a healthier future. This was achieved by integrating both qualitative and quantitative data analyses Figure 2 summarises the research blueprint.

Each method employed is discussed in greater detail in the subsequent sub-sections, providing a comprehensive understanding of the techniques integral to our study.

### 3.1. Community Engagement and Expert Interviews

To better understand the perspectives and experiences of individuals impacted by air pollution caused by the nearby copper mine, a series of three (3) focus groups were conducted in Kankoyo. This township has been directly affected by mining operations. A purposive sampling technique was used to select a sample of 24 residents from various places within the community, guaranteeing inclusive representation. The participants were chosen based on their varying ages and backgrounds, as indicated in Table 2. Clinic 5 Community-Based Volunteers (CBVs) facilitated this process. Subsequently, these volunteers assisted in the allocation of participants into three groups, each consisting of eight (8) participants, each with a particular subject. The group discussions were held at the local facility Clinic 5. Group 1 examined the self-reported respiratory problems caused by pollution, Group 2 analyzed the effects of pollution on respiratory health among various age groups, and Group 3 expressed their opinions on pollution levels and their variations across Kankoyo. After obtaining the participants’ informed consent, we conducted conversations using a semi-structured interview guide in the languages they preferred, which were Bemba and English. We also interviewed two experts who knew the current challenges faced by Kankoyo. These individuals included a clinic officer from Kankoyo Clinic 5 and a senior environmental specialist from the district health office (Table 3). The purpose of conducting these interviews was to gain further insight from both a clinical and a regulatory perspective. The qualitative data obtained from focus groups and expert interviews was subjected to thematic analysis using the NVivo software tool, and the data were transcribed verbatim. To ensure the accuracy and reliability of our findings, we employed a triangulation approach by incorporating data from both focus groups and expert interviews. Additionally, we diligently maintained a comprehensive audit trail that documented various aspects of our research, including the research process, interview guide, coding decisions, and data analysis procedures.

### 3.2. Spatial Analysis

#### 3.2.1. Hotspot Analysis

**Figure 3:**
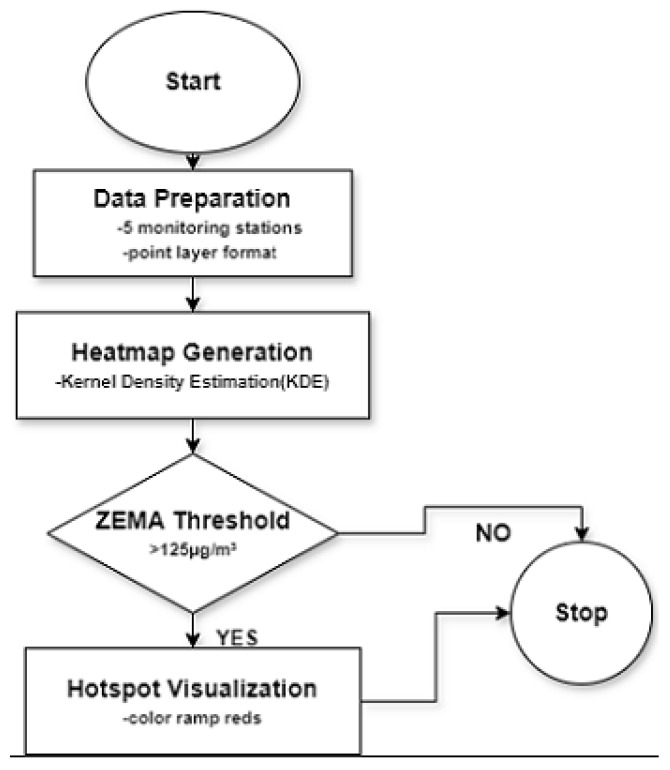
Summary of the spatial analysis methodology.

The proposed methodology for conducting a spatial hotspot study on sulphur dioxide (SO${\;\;}_{2}$) levels in Mufulira mine areas involved the identification of hotspots as areas that exceed the 24 h ambient SO${\;\;}_{2}$ guideline limit of 125 μg/m${\;\;}^{3}$ as established by the Zambia Environmental Management Agency (ZEMA) [26]. The selection of this criterion was based on its potential adverse effects on respiratory well-being. We obtained the SO${\;\;}_{2}$ data measurements we used for this analysis from Mopani copper mines through a study by [25] for the five monitoring points in 2020: Kankoyo Clinic 5, Butondo, Kantanshi Clinic 3, Kantanshi Clinic 5, and Malcolm Hospital. The data were then converted into a point-layer format without further cleaning steps. The heatmap was generated via the Heatmap (Kernel Density Estimation) plugin within the QGIS software. Kernel Density Estimation (KDE) is a well-known algorithm used to examine the spatial arrangement of point data, and a heat map provides a clear visual representation of the spatial patterns of points calculated using KDE, ensuring that there are no overlaps in the visualization process [27,28]. The visualization was employed to emphasize regions exhibiting heightened emissions within a 1000 m radius of each station, thus demonstrating the spatial arrangement of SO${\;\;}_{2}$ concentrations. Hotspots were identified by analyzing the heatmap, where places with a concentration of 125 μg/m${\;\;}^{3}$ or more were classified as hotspots. This indicated that individuals living in those areas faced increased risks of inhalation exposure. The heatmap utilized colour ramp reds to highlight increased amounts of SO${\;\;}_{2}$ and was overlaid onto a base map to assess the pathways by which nearby residents are exposed. The study used QGIS and its Heatmap plugin, with the simplified algorithm shown in Figure 3.

#### 3.2.2. Wind Profile for Kankoyo

To understand wind characteristics in the study area, Kankoyo, data from the ZEMA Kankoyo Clinic 5 monitoring point, spanning three years (2021–2023), were collected for wind direction (in degrees) and wind speed (in m/s). Initial preprocessing in Microsoft Excel involved data cleaning to remove outliers and erroneous values. Wind direction measurements were categorized into discrete compass directions (e.g., N, NNE, NE) using nested CHOOSE formulas. A pivot table summarized the frequency of each wind direction category, providing a preliminary understanding of wind patterns. For advanced analysis and visualization, the preprocessed data were exported to Python. Using the pandas library, the data were organized into a data frame for efficient manipulation. Frequency analysis quantified the proportion of winds from each direction and their associated speeds. A combined windrose diagram was generated using matplotlib and windrose libraries, illustrating the distribution of wind directions and speeds with different colours representing various speed ranges.

### 3.3. Retrospective Data Analysis

The retrospective data analysis research methodology guided this part of our study in the attempt to establish a relationship that may exist between SO${\;\;}_{2}$ exposure and respiratory illnesses over 15 years in Kankoyo Township. Using data science techniques, the analysis followed a data-driven perspective to identify the trends in respiratory health outcomes alongside the respective differences in levels of sulphur. The monthly statistics for health records covering the period of 2009 through 2023 were specially obtained from the Provincial Health Office Ndola, where the cases of respiratory illness are documented together with demographic information such as age. On the other hand, SO${\;\;}_{2}$ concentration for the same period was obtained from the Zambian Environmental Management Agency (ZEMA) for the Kankoyo Clinic 5 monitoring point. The datasets underwent pre-processing through merging and the subsequent treatment of missing values and feature engineering for a better understanding of the datasets. Exploratory Data Analysis (EDA) was then carried out by visualizing the temporal patterns of SO${\;\;}_{2}$, looking for trends and relationships with cases of respiratory illnesses. Correlation analysis was also performed to find some relationship between the two datasets. The study also examined the significance level of the association between exposure to SO${\;\;}_{2}$ and respiratory illness. Subsequent time-series analysis was undertaken to gain insights into the trends and patterns seen over the previous 15 years. A valuable understanding was gained of the relationship between respiratory health and ambient sulphur levels in Kankoyo.

## 4. Results

### 4.1. Community Engagement Findings

The qualitative data collected from focus group conversations in Kankoyo were subjected to thematic analysis after the community engagement events. The process of qualitative data analysis entailed exploring individual experiences, thoughts, interpretations, convictions, principles, and emotions about the topics of air quality and mining operations within their locality. The use of coding techniques enabled researchers to effectively organize and evaluate the data [29].

#### 4.1.1. Thematic Maps

**Figure 4:**
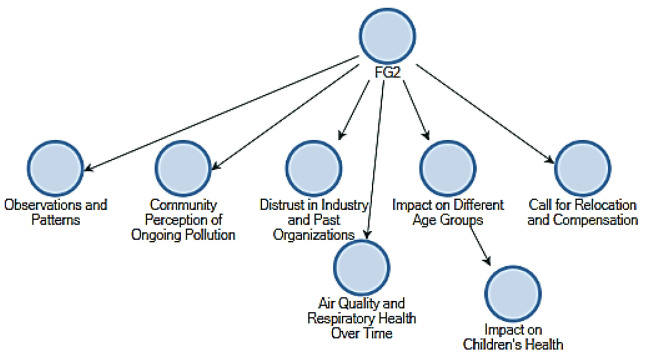
Focus group 2.

**Figure 5:**
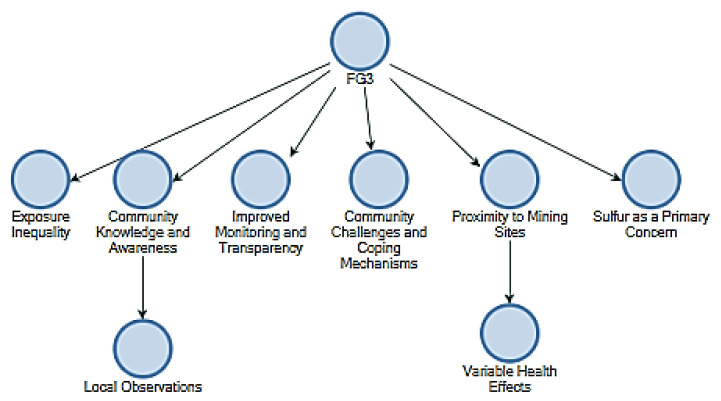
Focus group 3.

**Figure 6:**
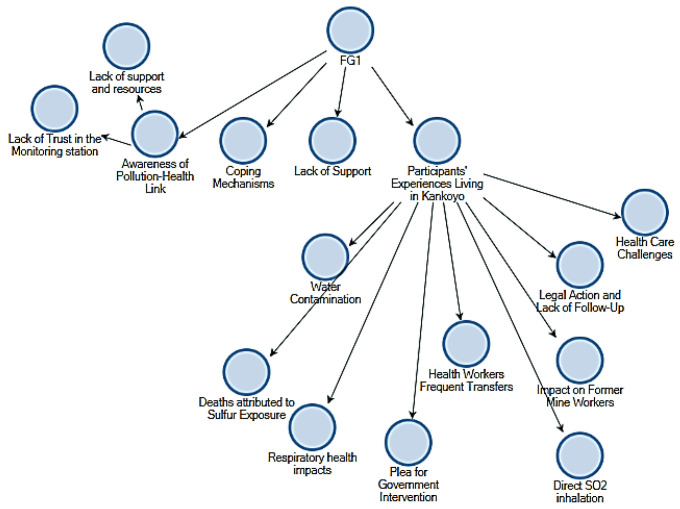
Focus group 1.

The initial stage was conducting a first-order coding process, wherein primary and secondary themes were found through thematic analysis. Thematic maps denoted as FG1, FG2, and FG3, were generated using the NVivo software to provide a more comprehensive summary of the topics identified after each focus group session. The maps played a crucial role in the initial stages of organizing the findings derived from the community engagement sessions. Figure 4 (FG2) provided the correspondence of pollution perception and indicated that the community’s age groups were deferentially affected. The sub-themes that supported the main theme included historical observations that generations of people in the society had noticed over time concerning pollution patterns, distrust by the people towards the regulating bodies, and resultant calls for remedial measures including relocation and compensation for the affected populations. Figure 5 (FG3) showed the painted limits of salient features: community awareness and response, with inequality in exposure to pollution. The theme overall was the variation with which pollution affected community members, underlying sub-themes that included local observations, coping strategies for pollution, and demands for better monitoring and transparency. This map reflected a community that advocated for equitable treatment and proactive engagement in pollution management. Figure 6 (FG1), this was presented as the immediate and lived experiences of community members about pollution, focusing on the health implications they faced. The core theme was found to be direct impacts on health, while the sub-themes touched on things like people’s problems in accessing health care and reported incidences of respiratory conditions related to exposure to sulphur. The sub-themes also gave a collective call for help to reflect on the critical need for health interventions and support systems that were expressed.

#### 4.1.2. Aggregated Themes

The analysis proceeded directly from the generation of thematic maps that summarized the first-order codes to defining second-order codes, which summarized the themes effectively and saved as aggregated themes. This involved a comprehensive review of the themes identified to draw clear parallels and linkages. These cohesive themes were then directly used to allow an efficient extrapolation of the overall patterns evident from within the qualitative data.

##### Focus Group 1

(a) Health Impacts of Air Pollution;
  Participants deliberated on the adverse health consequences of air pollution, including weakened immune systems, respiratory illnesses, and TB prevalence, with some mentioning the tragic death of an infant due to sulphur exposure. One participant recounted, stating,

“If you already have bronchitis or asthma, when you inhale sulphur dioxide, the cough worsens and can become persistent. Some people who have never had a serious cough still experience continuous coughing, resembling flu symptoms. It’s especially distressing for babies, I remember a case in Section C where a baby died under similar circumstances.”

(Speaker 3, female)

Another speaker added:

“Sometimes there are situations when sulphur is released, and then when you inhale, you experience serious chest pains.”

(Speaker 2, Male)

Another resident shared a particularly impactful case of sulphur dioxide exposure she experienced in 2018. While walking with friends on Fibusa grounds, directly opposite the acid plant in Kankoyo, she inhaled a high concentration of sulphur released from the plant. She described experiencing immediate fainting and chest pains later confirmed by medical reports from Malcolm Moffat Hospital as respiratory distress linked to the exposure. Despite this documented case, the participant reported receiving no further support, highlighting potential gaps in accountability and community support mechanisms. (Speaker 8, Female)

(b) Inadequate Healthcare and Support;
  Challenges accessing healthcare were highlighted, including medication shortages, discrimination at clinics, and inadequate support for chronic conditions. Speakers recounted their experiences:

“Even when you go to the clinic or hospital, they will just brush it off, especially if you mention ’Kankoyo’ at the end they won’t care at all.”

(Speaker 2, Female)

Additionally, another participant made an observation:

“Sometimes there are situations where medicine, let’s say for those of us who frequently experience respiratory blockages or have conditions like asthma, should always be available. Why should there be shortages of medication?”

(Speaker 3, Female)

(c) Environmental Damage and Livelihood Difficulties;
  Beyond health concerns, participants discussed environmental damage, such as damaged crops and houses, impacting their ability to sustain livelihoods:

“Here in Kankoyo, we’ve truly suffered. If you look around, we don’t have the opportunity to cultivate anything because our land is destroyed. Unlike our friends in townships like Kamuchanga and others, who can at least grow something like kalembula and chibwabwa in their backyard gardens, here, it’s a different story,”

(Speaker 7, Female)

Observed one speaker.

(d) Distrust and Frustration with Authorities;
  There was a general sense of distrust in the government and the mining company, with participants questioning the accuracy of monitoring and transparency in decision-making. One speaker expressed skepticism:

“Sister, to tell the truth, I don’t trust the readings from this monitoring station. In my opinion, they’re not accurate because if they were, there would have been follow-up actions. They’re giving the government incorrect readings. I recall a time when the government (ZEMA) brought their monitoring station, and mysteriously enough, sulphur emissions ceased when they arrived.”

(Speaker 4, Male)

(e) Coping Mechanisms and Resilience;
  Despite difficulties, participants shared coping mechanisms like utilizing home remedies and seeking assistance within the community. Some participants recounted:

“We’ve become accustomed to it. When sulphur is released, there’s nowhere to escape as long as you’re breathing. Everything in this area is affected. Sometimes, you just have to lock yourself indoors, but you’ll still smell it.”

(Speaker 4, Male)

“As for me, I used to carry Colgate and strong cough sweets. The fresh scent and the sensation of breathing in deeply would make me feel better. Sometimes, even Vicks would provide relief.”

(Speaker 7, Female)

##### Focus Group 2

(a) Declining Air Quality and its Impact on Health;
  Participants expressed concerns about deteriorating air quality and its link to respiratory illnesses, highlighting the invisible nature of sulphur emissions.
  One participant noted,

“It’s just the same. In fact, it has become worse. We used to see sulphur being released a long time ago, but now it comes hidden, very powerful, very strong, even affecting our chests without us seeing what has hit us.”

(Speaker 1, Male)

Another participant shared their personal experience, saying,

“As for me, I first started feeling sick in 2014, but it has gotten worse now. On December 13th, 2023, I was discharged from the hospital after being admitted due to chest pains.”

(Speaker 5, Male)

(b) Impact on different age groups;
  Participants emphasized that age was not a determining factor in how pollution affected them. One remarked:

“There is nothing like being old or young, everyone gets affected. Where can one go because if it’s a baby, it’s a problem throughout?”

(Speaker 2, Male)

Another participant echoed this sentiment, stating,

“As long as you are breathing, you are supposed to get affected. There is nothing like being old or young, everyone gets sick.”

(Speaker 1, Male)

A mother shared her emotional experience with her 6-year-old boy, expressing the challenges they face due to pollution-related health issues, saying,

“After he was born, we stayed a bit, and then he started experiencing blockages just like this because we used to stay on this side of Kankoyo, section C. So when sulphur gets released, sometimes he would briefly die, only to wake up in the hospital with oxygen. He is not okay in the chest. He was later diagnosed with asthma after several tests.”

(Speaker 8, Female)

(c) Community Engagement and Desire for Solutions;
  Despite challenges, the community remained engaged and hopeful for improvement, calling for solutions and better healthcare access.
  One participant suggested,

“Government, the health minister, and the ministry of mines should sit down together to find a new place because we are in danger here.”

(Speaker 1, Male)

Another participant emphasized the urgency of relocation, stating,

“Yes, relocating us to another area is necessary because otherwise, we will continue to fall ill. This entire place should be designated as a mining zone.”

( Speaker 5, Male)

On the contrary, another participant voiced a different concern, saying,

“My main complaint is that even if we don’t relocate, they should provide us with money and food. When someone is sick and lacks food, their illness will only worsen.”

(Speaker 3, Male)

##### Focus Group 3

(a) Unequal Exposure and its Consequences;
  Participants discussed the unequal distribution of pollution exposure, with those near mining sites experiencing higher levels of pollution and health concerns.
  One ex-miner recounted,

“It’s just the same because when you leave the mine, you inhale something else outside. It even becomes worse sometimes. As you leave the mine, you find sulphur has already been released on the surface. So there is no difference; it’s just the same everywhere.”

(Speaker 1, Male)

Another speaker observed,

“Those who are closer to the mine, the areas are dangerous because even just the explosives, when they start them, you just feel as though you will be buried underground. So that’s why they say people who reside on the other side should relocate because the area is not good.”

(Speaker 2, Female)

Furthermore, another speaker added,

“When sulphur is released, it comes with power, especially to us who are very close. You find that for those who live further away from the mine, by the time sulphur reaches their area, it would have lost its strength. While you, who are close, would have already inhaled a lot. So that’s the difference that is there.”

(Speaker 3, Female)

Distrust in monitoring and transparency: Deep distrust in official monitoring efforts was expressed, with demands for independent verification and transparency. One participant observed:

“That is why people from government (ZEMA) let’s also put it this way: we compare if indeed these readings we are given are correct, you see? So, this thing they have mounted here is only meant to blind us.”

(Speaker 4, Male)

Another participant expressed shock and made this observation:

“Now see, sister, this is why I laugh at the government. The situation was supposed to be that once the mine comes to collect their readings, they also carry along someone from the community-based volunteers to witness and know the truth, you see? There is no one that they carry along, they just check the readings alone. So even when they discover that the readings are high, they won’t tell anyone; they check alone. Once they come out, their response is, everything is okay.”

(Speaker 5, Female)

(b) Community Challenges and Coping Mechanisms:
  The community faced challenges such as a lack of knowledge about mitigation tools and living with uncertainty, leading to protests and demands for better support and education initiatives.
  One participant lamented:

“So now, because of their lies, that is why you see our children; once they get upset, they start rioting. This sulphur has caused deaths for residents; a newborn baby once died just after being exposed to sulphur. Someone is walking suddenly they die, so we start riots here. It’s just now that we have slightly behaved ourselves, sometime back it was bad.”

(Speaker 6, Female)

#### 4.1.3. A Comparative Analysis of Three Kankoyo Focus Groups

We compared themes from three Kankoyo focus groups to reveal both shared concerns and individual experiences regarding air pollution. Themes included health impacts, information trust, support gaps, environmental damage, coping mechanisms, specific worries, and demands. The comparison provided a lucid depiction of the common challenges and varied encounters of Kankoyo residents grappling with the repercussions of air pollution on their daily existence. A prevalent apprehension over respiratory ailments, anxieties regarding potential long-term health consequences, and a dearth of confidence in air quality data underscored the gravity of the matter. The concerns encompassed not only health-related issues but also various obstacles such as restricted healthcare accessibility, environmental degradation, and the coping mechanisms employed by individuals in the face of dissatisfaction. While there was a shared demand for improved healthcare accessibility, divergent perspectives on relocation and the necessity for increased transparency regarding environmental matters were evident, highlighting the multitude of viewpoints within the community (See Table 4).

### 4.2. Expert Interview Findings

To obtain a solid understanding of pollution’s impact on Kankoyo Township, Zambia, we interviewed healthcare and environmental management specialists. These interviews were conducted with a Clinic Officer from Clinic 5 Kankoyo and a Senior Environmental Health Specialist from the District Health Office (DHO) Mufulira. The main aim of this approach was to harmonize the community views with professional insight through the use of semi-structured interviews for a comprehensive understanding of the air pollution problems and possible solutions within Kankoyo.

#### 4.2.1. Clinic Officer

1. Healthcare Operations and Data Collection
  Clinic 5 is available on a 24/7 basis and structured into departments and data collection procedures. And transition to digital systems, like Smart Care, is on the way for enhanced data management. Limitations in advanced diagnostic equipment necessitate collaboration with external facilities.

“We lack the necessary equipment for testing. We don’t have the tools, we don’t have enough resources. We have to rely on the lab. Yes, these are some of the challenges. So, you often end up sending your patients to the hospital for tests, but they don’t always return with the results. Sometimes it’s because they lack transportation. So, they resort to buying over-the-counter drugs like Amoxil and Panadol and self-medicate. Then, months later, they return when their condition has worsened.”

2. Environmental Factors and Respiratory Health
  While directly attributing health conditions to specific pollutants is challenging, the Clinic Officer acknowledges the potential impact of SO${\;\;}_{2}$ emissions from mining activities on respiratory health.

“When it comes to treatment, pinpointing respiratory illnesses solely due to sulphur dioxide exposure is a challenge. Diagnosing conditions based solely on this exposure is difficult, ideally, we should be relying on diagnostic equipment to distinguish between ailments like pneumonia, TB, bronchitis, or whooping cough, but unfortunately, we lack such equipment. As a result, clinical treatment is typically based on symptoms.”

3. Reduction in Asthma Cases
  A perceived decrease in asthma cases was observed after the installation of air quality monitoring equipment by the mining company.

“There has been noticeable improvement and change. Before the installation of the equipment, we saw a high influx of patients, particularly those with asthma, a common respiratory condition exacerbated by risk factors prevalent in mining areas. The number of cases was overwhelming. However, since the installation of the equipment, the frequency has significantly decreased. Previously, we could see up to 10, 15, or even 20 cases in a day, but now it’s only one or two. This shows a significant improvement that the new equipment has made possible by facilitating better control over sulphur dioxide emissions.”

4. Respiratory Cases Linked to Environmental Pollution
  An estimated 75% of treated respiratory cases are potentially linked to environmental pollution, primarily SO${\;\;}_{2}$ emissions.

“About three-quarters of the cases are related to respiratory tract infections, likely linked to the mining area mostly sulphur. However, there are also cases of malaria and diarrhea, but respiratory tract infections are predominant in the area.”

#### 4.2.2. Senior Environmental Specialist

5. Data Sharing and Monitoring

  - Mopani provides certain emission-related information upon request, but routine data sharing of all pollution points is not standard practice.

“But they don’t share that information proactively; we have to specifically request it. However, upon request, they can provide us with the necessary data.”

  - Real-time monitoring of air emissions allows continuous tracking of pollution levels:

“Yeah, Mopani has more than one monitoring station. We have one at clinic 5, and there are others at different clinics.”

  - While SO${\;\;}_{2}$ is a primary focus, other potential relevant pollutants could exist.

“To the best of my knowledge, there may be other particulate matter such as solids or liquids, but what I know for certain is sulphur.”

6. Mitigation Measures and Community Engagement

  - The establishment of emergency trays for individuals with asthma reflects a reactive approach to managing health impacts.

“There has been some concern regarding the health and safety of the city, particularly for individuals with conditions like asthma. The company had previously established numerous emergency trays in various communities, especially when sulphur was released. However, this time around, they haven’t done so. I believe emergency trays are still available for asthma patients exposed to sulphur, facilitating easy access to treatment. This initiative was implemented before the smelter plants were upgraded.”

  - The environmental specialist further noted that monitoring stations contributed to emission control and adherence to established thresholds.

7. Commitment to Continuous Improvement

  - The Senior Environmental Officer emphasized the need for ongoing improvement in mining operations to minimize environmental and health impacts.

“So together, we can manage to control and prevent the spread of these high-efficiency pollutants. Ultimately, the goal goes back to Mopani to continuously improve its operations. At least there have been reductions both from the health and environmental perspectives.”

Upon completion of the analysis of the expert interviews, which aimed at complementing community findings, we continued to triangulate the two sets of findings. The notable aspect of Table 5 lies in its adept integration of community voices and professional perspectives, thereby providing compelling evidence for the potential correlation between SO${\;\;}_{2}$ inhalation and the respiratory ailments described by the residents of Kankoyo. The connection was strengthened by the congruence between community apprehensions over night-time pollution emissions, individual encounters, and expert research on the detrimental effects of SO${\;\;}_{2}$ exposure. The table additionally identified crucial areas that necessitate prompt action. Inadequate healthcare accessibility and difficulties in data gathering hindered the effectiveness of treatment and a comprehensive comprehension of the situation. Phenomena like land degradation and limited agricultural activities served as examples of environmental degradation that go beyond human health. This highlighted the urgent need to implement mitigation measures aimed at protecting both public health and the environment.

## 5. Spatial Analysis—Findings

### 5.1. Hotspot Analysis

**Figure 7:**
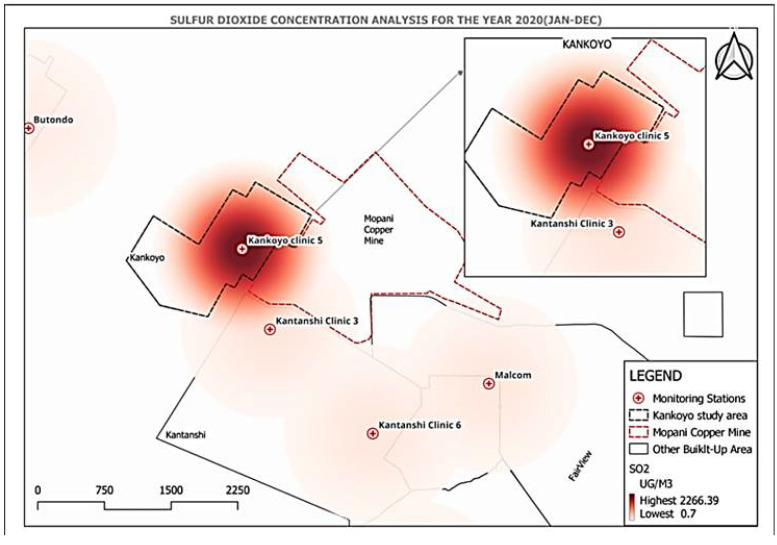
Spatial distribution of SO${\;\;}_{2}$ concentrations in Mufulira.

Two probable SO${\;\;}_{2}$ hotspots were found within the mine region, namely Kantanshi Clinic 3 and Kankoyo Clinic 5, based on our spatial analysis. The area of concern that stood out was Kankoyo, which surpassed the ZEMA threshold by a significant margin of 1713.11% at a maximum concentration of 2266.39 μg/m${\;\;}^{3}$. Kantanshi Clinic 3, situated at a distance of roughly 894 m from the mines, demonstrated lower concentrations in comparison to Kankoyo Clinic 5, which is located at a distance of 650 m. Data from five monitoring stations were spread out across the mine area, which showed very low concentrations at greater distances, supported the aforementioned trend. This spatial distribution, with Kankoyo experiencing significantly higher SO${\;\;}_{2}$ levels compared to Kantanshi Clinic 3 and other stations, raised concerns about the potential for increased direct inhalation of harmful pollutants, particularly for those residing or spending time closer to Kankoyo Clinic 5. Figure 7 visually depicts the spatial SO${\;\;}_{2}$ distribution with a colour gradient. Dark red represented the peak concentration around Kankoyo Clinic 5, transitioning to lighter shades and lower concentrations as you move further away. This highlighted the dramatic exposure difference between areas close to the mine and those farther away. Interestingly, variations were observed within Kankoyo itself. While the Kankoyo Clinic 5 area exhibited the highest concentration, Butondo, a neighbouring compound, had significantly lower levels due to its greater distance from the pollution source. This observation continued to show the importance that must be placed on proximity and wind patterns in gauging the distribution of SO${\;\;}_{2}$ over this particular geographical area of concern. Although specific individual levels of inhalation were not available, the spatial observations gave very clear indications of the likelihood of risk to residents in Kankoyo, especially near the hotspot area. High concentrations of SO${\;\;}_{2}$ told of its higher possibility for direct inhalation than those regions, which were further away. It was, therefore, a question of the potential future respiratory health effects, especially for residents and people who spend long periods near the area with high concentrations. Our findings supported the description regarding continued concern from past researchers, as well as community engagement, and the fact that the people closest to the mine continued to experience the most severe air quality. Experts classified Kankoyo as being severely contaminated, even though pollution levels had decreased compared to previous years. The multi-pronged strategy delivers compelling evidence of the health risks posed by high levels of SO${\;\;}_{2}$ and therefore calls for urgent intervention.

### 5.2. Wind Profile for Kankoyo

**Figure 8:**
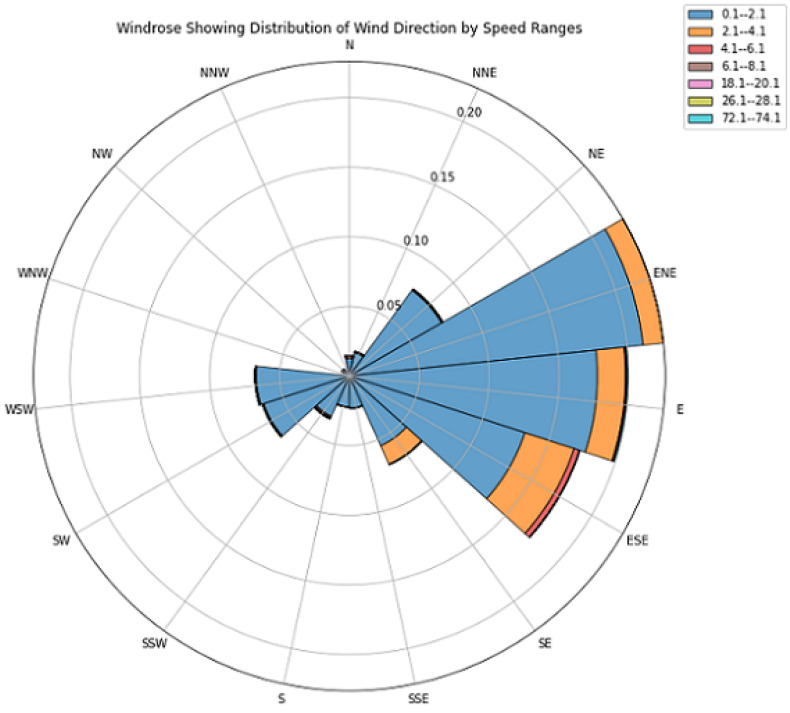
Prevailing Wind Directions and speeds in the study area, Kankoyo, Mufulira.

The analysis of wind patterns in Kankoyo from 2021 to 2023, shown by the windrose diagram (Figure 8), gives us important information about the area’s wind conditions. The data show that winds mostly blow from the east-northeast (ENE) and east (E) directions, usually at speeds between 0.1 and 2.1 m/s (indicated in blue). This pattern aligns with the location of the smelters relative to Kankoyo, suggesting a likely pathway for SO${\;\;}_{2}$ and other pollutants to reach the residential area. The windrose diagram uses different colours to represent various wind speeds. Each spoke indicates a wind direction, while the colours within each spoke show different wind speeds: blue (0.1–2.1 m/s) for the most common wind speeds, orange (2.1–4.1 m/s) for moderate wind speeds, and other colours for higher wind speeds, which are less common. This colour coding helps us understand how wind speeds affect the movement of pollutants from the Mopani Copper Mine Smelters’ direction. The frequent occurrence of low speed winds (0.1–2.1 m/s) raises concerns about the potential for limited dispersion and accumulation of pollutants within Kankoyo. This could increase the exposure of residents to SO${\;\;}_{2}$, a known respiratory irritant, and potentially contribute to the observed link between proximity to the mining site and respiratory illnesses. Although less frequent, winds from the south-southeast (SSE) and south-southwest (SSW) directions also suggest other potential pollutant sources affecting air quality in Kankoyo. Analyzing these wind patterns is a key part of the broader mixed-methods investigation. By understanding the prevailing wind conditions and their potential role in transporting pollutants, this study strengthens the evidence linking SO${\;\;}_{2}$ exposure from the Mopani Copper Mine Smelters to the higher rates of respiratory illnesses in Kankoyo. These findings highlight the importance of including meteorological data in health studies, especially in areas impacted by industrial activities. To further visualize the spatial context of these wind patterns within the Mufulira district, a map depicting the prevailing ENE wind direction and its potential impact on surrounding areas can be found in the (Supplementary Material Figure S1).

## 6. Retrospective Data Analysis-Findings

In an era characterized by the significant influence of data-driven insights on the development of public health and environmental policy [30,31], this portion of our findings concern a comprehensive retrospective analysis spanning 15 years to examine the air quality and health records of Kankoyo. The main objective of our study was to investigate the complex correlation between exposure to sulphur dioxide (SO${\;\;}_{2}$) and the incidence of respiratory diseases. With the utilization of data science techniques and Python programming, we initiated a comprehensive quantitative endeavor that involved the gathering of data, their preprocessing, exploratory analysis, and rigorous statistical scrutiny. The scope of this analysis was limited to addressing two primary sub-research inquiries:

(a) What correlations exist between variations in sulphur dioxide (SO${\;\;}_{2}$) exposure levels and the incidence of respiratory symptoms among residents of Kankoyo?
(b) What trends or patterns are observed in the prevalence of respiratory illnesses in Kankoyo over time and corresponding levels of SO${\;\;}_{2}$ exposure?

### 6.1. Temporal Trends and Patterns

To uncover the trends and patterns, we used the Python libraries Pandas and Matplotlib to study how sulphur dioxide (SO${\;\;}_{2}$) levels and respiratory illness cases changed over the past 15 years. We noticed ups and downs in both, suggesting possible seasonal changes or long-term trends. Monthly data showed variations in respiratory illnesses, hinting at seasonal patterns. We also used time series analysis to dig deeper into these trends, aiming to understand how SO${\;\;}_{2}$ exposure had affected respiratory health over time.

#### 6.1.1. Time Series Analysis

**Figure 9:**
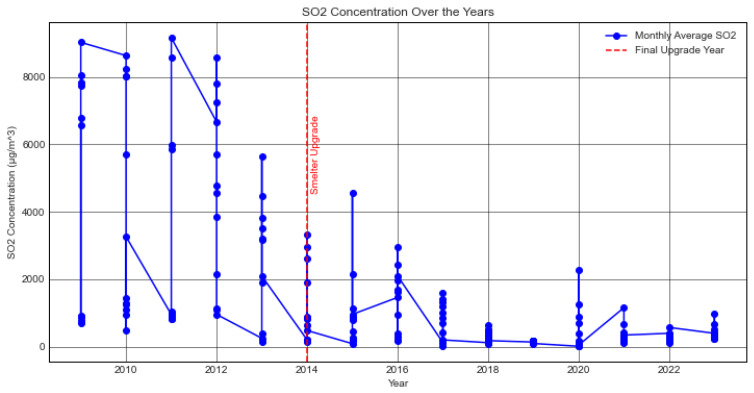
SO${\;\;}_{2}$ time series—2009 to 2023.

Figure 9 presents an analysis of the temporal patterns in sulphur dioxide concentrations spanning a period of 15 years, specifically from January 2009 to December 2023. The blue dots show the monthly averages of SO${\;\;}_{2}$ levels at the Kankoyo Clinic 5 tracking point, close to the mine acid plant. The lines that link these markers show the order of these measurements over time, showing how they have changed and grown from month to month over the last 15 years. The final upgrade phase of the Mufulira Smelter of Mopani Copper Mines Plc was completed in March 2014. The objective was to convert a minimum of 97% of the emissions of sulphur dioxide into sulphuric acid [32]. Before the implementation of the upgrade, the atmospheric concentration of sulphur dioxide consistently remained elevated, frequently above the ZEMA threshold of 125 μ/m${\;\;}^{3}$ for ambient air with a peak concentration just slightly over 8000 μ/m${\;\;}^{3}$, whereas the minimum value was 156 μ/m${\;\;}^{3}$. There was a noticeable improvement when comparing the period before the upgrade (2009–March 2014) with the period following the completion of the upgrade. The mean concentration of SO${\;\;}_{2}$ had a marked decrease of 81.5%, declining from 3564 μ/m${\;\;}^{3}$ to 660 μ/m${\;\;}^{3}$. Despite the implementation of the upgrade, instances of elevated concentrations persisted, particularly during certain months, albeit with reduced frequency and severity compared to the pre-upgrade period. This trend reflected ongoing efforts to reduce sulphur pollution, with improvements noted, especially complemented by the installation of monitoring stations around the mine area to monitor sulphur in the ambient air. It is imperative to bear in mind that despite the substantial reduction in emissions resulting from the improvement, the release of sulphur dioxide persists, albeit at a lower level compared to the pre-upgrade period. This aligned with the findings of both the clinic officer and the environmental specialist, who indicated that the enhanced smelter effectively managed sulphur emissions more comprehensively. However, the report confirmed that the community’s concerns about ongoing sulphur pollution were warranted since the pollution continued to occur, though at a reduced rate compared to before the upgrade.

**Figure 10:**
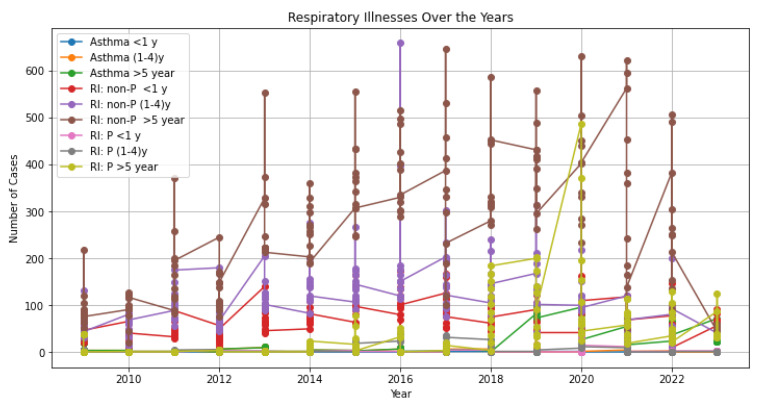
Respiratory illnesses time series—2009 to 2023.

Figure 10 shows the changes in respiratory illnesses at Kankoyo Clinic 5 over the past 15 years. The graph shows how sulphur pollution has affected the respiratory health of the community over an extended period. It presents the data in the form of a time series plot, with dotted average monthly respiratory cases categorized by age group and colour-coded accordingly. The respiratory cases the study looked at were asthma, non-pneumonia respiratory infections (RI: non-P), and pneumonia respiratory infections (RI: P) in people of different ages. The age groups were asthma <1 year, asthma (1–4) years, and asthma >5 years. The non-pneumonia respiratory infections groups were RI: non-P <1 year, RI: non-P (1–4) years, and RI: non-P >5 years and pneumonia, RI: P <1 year, RI: P (1–4) years, and RI: P >5 years. From 2009 to 2012, the number of respiratory illnesses stayed pretty low. At times, the number of non-pneumonia respiratory infections in the group over 5 years old went up, with some months seeing more than 300 cases. Surprisingly, this period coincided with high sulphur levels, showing that there may be a connection between the amount of pollution and health outcomes. After that, between 2012 and 2022, there was a clear rise in both non-pneumonia and pneumonia respiratory cases in most age groups, except for kids younger than 4 years old who had pneumonia. Peaks in cases were seen in 2016, 2017, 2020, and 2021, mostly involving kids aged 1 to 4 and individuals over 5 years old. At the same time, amounts of sulphur in the air also reached their highest points, mostly between 2009 and 2018, and also in some months of 2020. This further suggested a potential link between SO${\;\;}_{2}$ and respiratory illnesses. Before the smelter upgrade, there were not many cases of pneumonia, on average fewer than 50 per month. However, there was a big rise from 2018 to 2023, especially among individuals over 5 years old, with more than 100 new cases. In 2020, there were over 400 cases at their highest point, which happened at the same time that sulphur levels were high in some months. Even so, pneumonia cases were not as common as other respiratory illnesses, especially since sulphur levels were going up. From 2009 to 2017, the number of asthma cases stayed steady, mostly below 50 per month across all age groups. But from 2018 to 2023, there was a small rise, mostly among older age groups. This shows that sulphur pollution still harms lung health. Notably, the number of respiratory cases dropped significantly in 2023, which happened at the same time that sulphur levels dropped. These findings highlighted the complex relationship between sulphur pollution and respiratory health over the study period.

#### 6.1.2. Seasonal Variation Analysis

**Figure 11:**
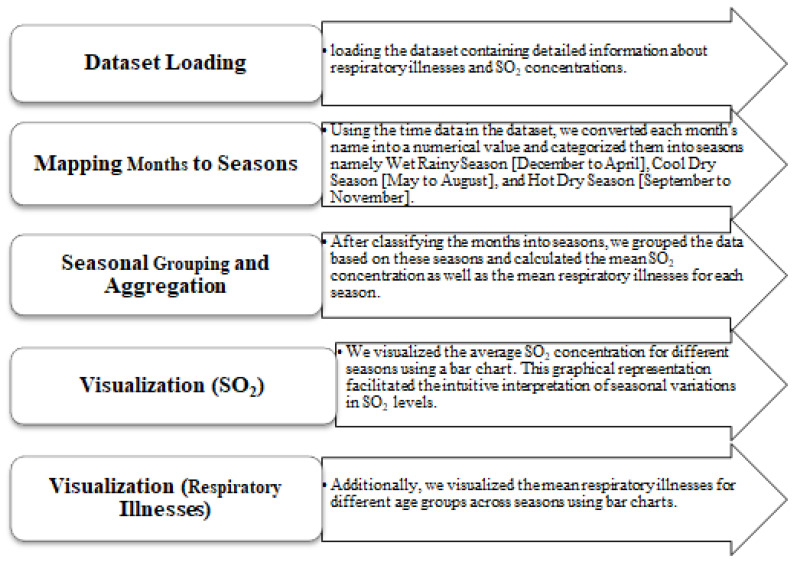
Feature engineering process description.

Seasonal variations were examined by employing feature engineering to gain a better understanding of the data. Significant fluctuations in atmospheric sulphur dioxide (SO${\;\;}_{2}$) levels and respiratory illness prevalence were observed in some months throughout the 15 years. As a result of the aforementioned trend, we categorized the months into seasons to enhance our comprehension of these transformations. A new field was added to the dataset to indicate the season of each month. This facilitated a more comprehensive examination of the impact of seasons on SO${\;\;}_{2}$ levels and respiratory illnesses. We employed feature engineering techniques to meticulously examine the seasonal variations and identify patterns and correlations that proved valuable for our research. The methodology employed to identify seasonal variations is depicted in Figure 11. This methodology facilitated our comprehension of the temporal dynamics between SO${\;\;}_{2}$ exposure and pulmonary well-being, hence enhancing the comprehensiveness and accuracy of our research.

##### Insights from Seasonal Variation Analysis

**Figure 12:**
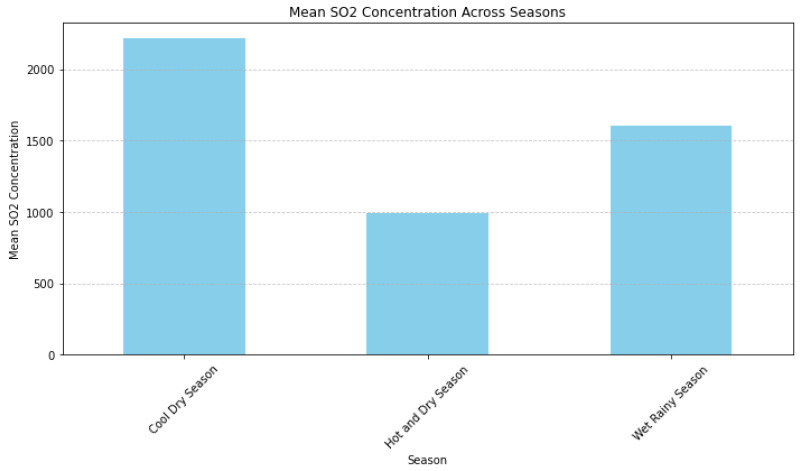
Mean SO${\;\;}_{2}$ concentration by season over 15 years.

**Figure 13:**
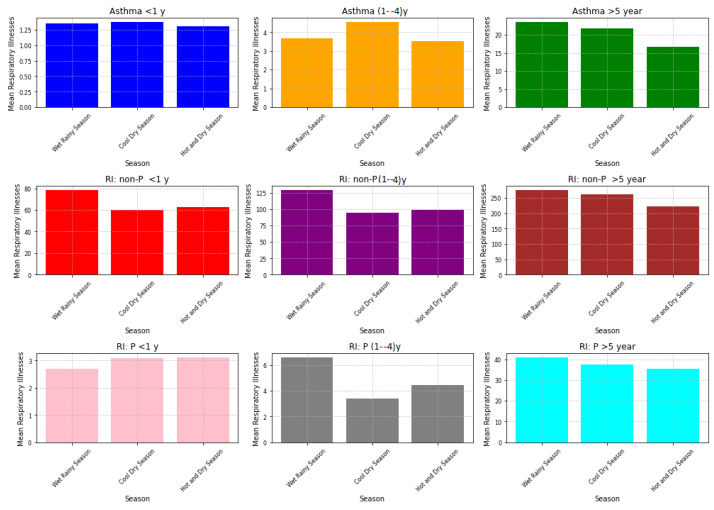
Mean respiratory illnesses by season over 15 years.

The bar graph in Figure 12 displays our 15-year data, indicating that the mean sulphur dioxide (SO${\;\;}_{2}$) concentration repeatedly reached its peak during the cool dry season. Following the wet rainy season, the hot dry season exhibited the lowest amounts of SO${\;\;}_{2}$. However, Figure 13 presents a detailed perspective on the occurrence of asthma in three different age groups: less than 1 year, 1–4 years, and above 5 years. These age groups were further classified based on seasons as wet rainy, cool dry, and hot dry. We further divided respiratory infections (RI), which includes both non-pneumonia (non-P) and pneumonia (P), into groups based on the same seasonal criteria as asthma. Non-pneumonia infections were put in row two, and pneumonia infections were put in row three.

The retrospective study of this seasonal variation indicated a complex correlation where elevated SO${\;\;}_{2}$ levels often corresponded with an increase in respiratory illnesses, most notably during the wet rainy season, across various age groups. The cool dry season trend was predominantly pronounced in children aged 1 to 4 with asthma cases, aligning with the season of peak SO${\;\;}_{2}$, suggesting a significant increase in asthma cases during this time. Asthma prevalence in infants under 1 year, however, remained relatively unchanged throughout the seasons, while those over 5 years experienced the highest incidences during the wet rainy season. In terms of respiratory infections, both pneumonia and non-pneumonia types were more common during the wet rainy season, with non-pneumonia infections showing a more consistent pattern across all age cohorts. In contrast, pneumonia infections displayed a subtler seasonal dependency, especially in the youngest cohort under 1 year.

These observations highlighted the intricate relationship between environmental pollutants and health outcomes. However, the effect of other factors such as cooler temperatures and viral outbreaks that happen during cool dry and wet rainy seasons could also be taken into account. These factors may make breathing problems worse, making it harder to blame SO${\;\;}_{2}$ alone. Thus, this evidence emphasized the need for more detailed research to untangle the complicated dynamics among various factors and their collective effect on respiratory health. The findings were further echoed by community concerns regarding heightened SO${\;\;}_{2}$ levels in certain seasons, such as cool dry and wet rainy seasons, stressing the critical need for continued research efforts in this domain.

#### 6.1.3. Correlation Analysis

This section explores the link between sulphur dioxide (SO${\;\;}_{2}$) exposure and respiratory illnesses among Kankoyo township residents from a quantitative point of view. Using Spearman’s rank correlation coefficient, a suitable method for non-normal distributions and ordinal data [33], we analyzed our data with Python’s SciPy and StatsModels libraries to help us measure the strength and direction of the relationships. By assessing *p*-values, we gauged the significance of observed associations; Table 6 presents the correlation findings along with their significance.

The results from our study in Table 6 shed light on how air pollution, specifically sulphur dioxide (SO${\;\;}_{2}$), interacted with community health, and they told an intriguing story about the community in Kankoyo. What stood out was the clear signal that older individuals over 5 years old were the most affected by SO${\;\;}_{2}$. This group showed a strong negative relationship with SO${\;\;}_{2}$ levels for asthma and both types of respiratory infections (pneumonia and non-pneumonia), indicating that as air pollution went up, so did these respiratory problems. This was not just a slight increase; the statistics (asthma (Spearman correlation coefficient, $\rho =-0.5090;p=3.4514\times 10^{-13}$), pneumonia ($\rho =-0.5734;p=4.9335\times 10^{-17}$), and non-pneumonia respiratory infections ($\rho =-0.3482;p=1.7869\times 10^{-06}$) showed that the link was too significant to ignore. For the youngest in the community, those under 1 year old, and somewhat older children, the situation with non-pneumonia respiratory infections presented a bit of a mixed bag. While the youngest and oldest showed a moderate negative correlation with SO${\;\;}_{2}$, meaning there was an evident impact, kids aged 1–4, on the other hand, did not seem to be affected in the same way because the significance was marginal. It was a curious finding that suggested how different bodies might react to the same environmental stressors at different ages. Interestingly, when we zoomed in on how SO${\;\;}_{2}$ levels might be affecting asthma in our little ones, those under 5, the concern seemed to dissipate. The data showed weak correlations that were not statistically significant (for instance, ρ=−0.0001 for infants with a *p*-value of 0.9986). Essentially for asthma in young children, SO${\;\;}_{2}$ did not appear to be a significant factor based on our current analysis. This was somewhat reassuring, but it did not lessen the overall concern about air pollution.

What did all this mean? Our exploration into how SO${\;\;}_{2}$ levels related to respiratory health revealed a complex narrative. The members of the Kankoyo community older than 5 years old were clearly at risk, with strong evidence pointing towards the need for authorities to address air quality to protect their health. Yet, the story changed as we looked at different age groups and different respiratory conditions, highlighting the complex manner in which the Kankoyo environment interacted with residents’ health. Thus, these results transcend academic interest, serving as a clarion call for action.

## 7. Discussion

In communities residing near mines, concerns regarding the potential adverse health effects of sulphur dioxide (SO${\;\;}_{2}$) in the air have been a historical battle. Our research examined the impact of SO${\;\;}_{2}$ on the health of the residents of Kankoyo, Zambia, with a particular focus on respiratory issues. We discovered fairly solid evidence that inhaling SO${\;\;}_{2}$ could be the cause of these problems, which underscored the critical need for us to continue our investigation and develop solutions.

In our focus group conversations, residents of Kankoyo expressed apprehensions regarding respiratory difficulties. Also discussed was a time when someone became gravely ill after being exposed to an excessive amount of SO${\;\;}_{2}$. This finding was unsurprising, given the established health risks associated with SO${\;\;}_{2}$ inhalation, as previously highlighted by scholars such as Axenhamn and Simukanga [20] and Mufumbi [25]. The community members were cognizant of the dangers and desired greater input and transparency regarding the air quality inspection process. Our findings supported what others had previously stated regarding the significance of open communication and community collaboration in addressing pollution [22]. Expert interviews with environmental and healthcare specializations agreed that SO${\;\;}_{2}$ could potentially exacerbate respiratory problems. Notably, the clinic officer noted a reduction in the incidence of asthma cases after the installation of air quality monitoring equipment by the mine. This finding underscored the significance of pollution control measures and potentially established a dose–response correlation. Yet this positive note also revealed a tough truth about the lack of enough healthcare resources in Kankoyo, highlighting the urgent need for better tools and information to tackle health issues head-on. Regarding the determinants of exposure levels, our spatial analysis substantiated the designation of Kankoyo as a region afflicted by SO${\;\;}_{2}$ pollution, where concentrations notably exceeded thresholds set by ZEMA. This analysis also examined prevailing wind patterns, which predominantly blow from the east-northeast (ENE) direction, where the Mopani Copper Mine Smelters are situated. This wind pattern further provided a plausible explanation for the elevated SO${\;\;}_{2}$ levels in Kankoyo. The importance of proximity to emission sources in other industrial contexts was consistent with the finding that proximity to the mining site was a crucial factor [10]. This called for a rethinking of how homes are built near such industries and how people’s exposure to pollution can be lessened.

The results of our retrospective analysis unveiled several noteworthy patterns. The potential correlation between heightened SO${\;\;}_{2}$ concentrations and a greater incidence of respiratory ailments merited additional investigation. The discernible seasonal trends identified, in which respiratory ailments reached their highest levels during periods of elevated SO${\;\;}_{2}$ concentrations and cool dry and wet rainy seasons, served as an initial reference for analyzing the intricate interplay between pollution and health consequences in Kankoyo. One salient discovery pertained to age-related sensitivities, as correlation analyses revealed considerably more robust correlations between SO${\;\;}_{2}$ and respiratory illness among senior age cohorts (>5 years). Although previous research had indicated that SO${\;\;}_{2}$ might have general respiratory effects [11,12], the age-specific vulnerability identified in Kankoyo introduced a fresh aspect that warranted further investigation in subsequent studies.

### Triangulation

The triangulation of findings obtained through the use of various methodological approaches improved the robustness of our study. This approach facilitated a comprehensive understanding of the intricate relationship between exposure to sulphur dioxide (SO${\;\;}_{2}$) and respiratory health in the region of Kankoyo. The methodology offered used various sources of information, including direct community engagement, geographical analysis, retrospective data examination, and expert presentations, to produce a comprehensive and diverse understanding of the subject matter.

A personal anecdote and the focus group discussions in the community highlighted the widespread concerns about respiratory issues. An individual who had a rapid loss of consciousness as a result of exposure to SO${\;\;}_{2}$ provided medical documentation that confirmed a diagnosis of respiratory distress. This particular scenario provided a compelling and pragmatic illustration of the detrimental impacts of sulphur dioxide (SO${\;\;}_{2}$). This narrative functioned as a significant intermediary, establishing a connection between local anecdotal information and clinical validation regarding the health hazards associated with SO${\;\;}_{2}$. Additionally, our examination of retrospective data yielded supplementary evidence, indicating a potential correlation between heightened levels of SO${\;\;}_{2}$ and the prevalence of respiratory ailments. The quantitative data served to validate the individual and collective concerns expressed during focus group deliberations while also providing statistical support for the observed health trends within the Kankoyo community.

However, the investigation uncovered a notable obstacle: the Kankoyo Clinic 5 healthcare facility, which served as the initial point of contact for affected individuals, sometimes lacked the essential diagnostic tools required to precisely identify disorders linked to exposure to environmental sulphur dioxide. Consequently, those with significant severity requiring advanced diagnostic abilities were directed to larger healthcare establishments, such as Malcolm Moffat Hospital. The noticeable disparity in diagnostic proficiency sharply contrasted with the definitive evidence of the adverse impacts of SO${\;\;}_{2}$ as recorded in Malcolm Moffat Hospital records, underscoring a fundamental deficiency in the healthcare system at the local facility. Spatial analysis enhanced our comprehension by pinpointing Kankoyo as a location with significant SO${\;\;}_{2}$ exposure. The analysis also noted frequent low-speed winds from the ENE, suggesting limited dispersion of pollutants and their potential accumulation in Kankoyo, contributing to the elevated levels observed. This spatial and meteorological evidence strengthened the argument for a link between exposure and respiratory health problems in the community. Healthcare and environmental experts endorsed the study’s conclusions, emphasizing the need to address the respiratory consequences associated with emissions.

The research technique employed in our study was triangulated, which involved the integration of qualitative community testimonies, quantitative analysis derived from retrospective investigations, clinical documentation, and expert perspectives. This methodology facilitated the provision of a comprehensive depiction of the health implications associated with SO${\;\;}_{2}$ pollution in the region of Kankoyo. The study presented an integrative approach that not only validated the notable correlation between exposure to SO${\;\;}_{2}$ and respiratory ailments, particularly in light of the community’s proximity to mining activities and prevailing wind patterns but also underscored the pressing necessity for enhanced healthcare infrastructure and focused public health interventions.

## 8. Conclusions

The study conducted in Kankoyo, Zambia, highlighted the considerable health hazards linked to the exposure of sulphur dioxide (SO${\;\;}_{2}$), particularly its impact on the respiratory well-being of individuals residing near mining activities. Through the utilization of community interaction, expert interviews, spatial analysis, and retrospective data review, a robust association was observed between heightened levels of sulphur dioxide (SO${\;\;}_{2}$) and heightened respiratory problems. This correlation was particularly evident during periods of peak exposure and among older demographics. We found significant gaps in the local healthcare system, particularly in primary care facilities in Kankoyo, even though new air quality monitors have made some improvements. The ongoing presence of SO${\;\;}_{2}$ still calls for stronger pollution control and better healthcare services. The local community has been vocal about needing more clarity and involvement in monitoring air quality. Our findings agree and highlight the ongoing need to explore how environmental pollution affects public health more deeply. Given all these factors, our study urgently recommended specific actions to address these issues:

1. ZEMA should implement a unified policy mandating that mining companies make real-time SO${\;\;}_{2}$ and other sources of pollution data accessible not only to ZEMA but researchers, affected communities, and the Ministry of Health via the district health offices. This data should be made accessible through user-friendly online platforms or public display systems to enhance transparency and inform policy decisions related to air quality and public health concerns.
2. ZEMA should invest in deploying low-cost air quality sensors as commended by the World Health Organization [1] and Chihana et al. [34] to alleviate financial burdens and supplement existing monitoring methods. Additionally, leveraging data science techniques to enhance data analysis offers deeper insights into air pollution patterns and their implications for public health.
3. The Ministry of Health should strengthen policies to ensure a consistent supply of essential medication for SO${\;\;}_{2}$ related respiratory conditions in Kankoyo. This would reduce the burden and health risk of residents relying on less-effective over-the-counter medications.
4. As a long-term measure, the ministry should explore the possibility of establishing specialized respiratory labs within Kankoyo Clinic 5 and other facilities around the mine area equipped with advanced diagnostic machines. This would improve diagnostic accuracy, reduce the number of required referrals, and enable data collection for research, monitoring, and providing targeted interventions.
5. A multifaceted informational campaign should be launched utilizing social media, websites, mobile applications, and community-focused platforms, developing engaging educational materials (videos, advanced visualizations through mathematical simulations, etc.) to increase understanding of the health dangers linked to SO${\;\;}_{2}$ exposure and enable people to take steps to prevent themselves.
6. Strong partnerships should be established between mining companies, universities, research centers, government agencies, and the Kankoyo community. This collaboration should focus on ongoing air quality monitoring, comprehensive health impact assessments, and the development of evidence-based solutions tailored to the local context.

In conclusion, the results from expert observations, community engagements, spatial patterns, seasonal trends, and age-related correlations all point to the need for more research. This research endeavored to fill a specific void in the existing body of literature regarding the localized consequences of SO${\;\;}_{2}$ exposure in the Copperbelt area of Zambia. 

## Figures and Tables

**Table 1:** Findings on mining-related air pollution, categorized by methodology.

Methodology	Study	Key Findings	Limitations
Literature review	[3]	Investigated air pollution (SO${\;\;}_{2}$, PM) from mining and its effects on health, plants, animals, and infrastructure.	Broad understanding of mine pollution effects on the environment from a literature point of view.
Mixed-Methods (Case Study, Qualitative, Quantitative)	[21]	Air, noise and water pollution, job losses.	Focused on corporate social responsibility.
	[10]	Explored the impacts of SO${\;\;}_{2}$ releases and discharge of mine waste on the air and water quality in Kankoyo Township.	The study did not investigate the potential health effects of SO${\;\;}_{2}$ on residents.
	[11]	Eighty-eight per cent of respondents in Kankoyo reported RTIs as a primary health concern attributed to SO${\;\;}_{2}$.	Focused on environmental risk management systems. No detailed findings to link health effects to SO${\;\;}_{2}$.
	[22]	Investigated the effect of mining on some parts of Kitwe and Mufulira.	The study was broad as it looked at various effects of mining on the environment in Kitwe and Mufulira.
	[23]	Pollution, dust, and heavy metals are harmful to the environment.	Focused on multiple pollutants.
Gas Concentration Measurement	[24]	SO${\;\;}_{2}$ and metals harm vegetation, wildlife.	Limited to one plant species and limited sampling points. No health impact analysis
Mathematical Modelling	[25]	Looked at mathematical models that depict the dispersion and concentration of SO${\;\;}_{2}$ emissions from specific mining firms.	The study focused on modeling sulphur movement in mining areas using mathematical approaches
Hedonic Pricing and Contingent Valuation Method	[12]	Residents reported various health issues, including fatalities, attributed to air pollution in Kankoyo (SO${\;\;}_{2}$ emissions).	Investigated the correlation between SO${\;\;}_{2}$ emissions and their impact on residential property values in the Kankoyo.

**Table 2:** Focus group participants.

Group	Demographics	Participant Code	Age Range
*FG1*	Mixed (4 females, 4 males)	FG1-SP1–FG1-SP8	Age range: 20–50s
*FG2*	Mixed (2 females, 6 males)	FG2-SP1–FG2-SP8	Age range: 6–70s
*FG3*	Mixed (3 females, 5 males)	FG3-SP1–FG3-SP8	Age range: 20–50s

**Table 3:** Expert Interviewees.

Interview	Participant ID	Affiliation	Expertise
*EX1*	EX1-EP1	Kankoyo Clinic 5	Clinic Officer
*EX1*	EX2-EP2	District Health Office	Senior Environmental Specialist

**Table 4:** Comparative analysis matrix: key findings across focus groups.

Theme	Focus Group 1	Focus Group 2	Focus Group 3	Connection to Research Question
*Health Impacts*	Severe respiratory problems (coughing, chest tightness, asthma) impact all age groups. **Individual Story**: A resident experienced immediate fainting after inhaling sulphur. Medical reports confirmed exposure and diagnosed respiratory distress.	Emphasized worsening conditions, highlighted hidden pollutants causing internal damage, and mentioned the death of a newborn attributed to sulphur exposure.	Mentioned silica dust exposure near the mine, focusing on cumulative effects such as damaged houses and infrastructure.	Direct inhalation of SO${\;\;}_{2}$ can exacerbate respiratory problems, as evidenced by the resident’s case. Hidden pollutant exposure (mentioned by other groups) could pose additional health risks, warranting further investigation.
*Distrust & Information*	Doubts about air quality monitoring data accuracy, suspicion of manipulation by the mining company.	Mentioned past organizations promising help without action.	Highlighted distrust in the monitoring station and described witnessing night-time releases to evade detection.	Lack of trust in monitoring data could obstruct understanding of SO${\;\;}_{2}$ exposure levels and risks.
*Lack of Support & Resources*	Inadequate healthcare access and medication shortages, discriminatory treatment at facilities.	Mentioned trouble obtaining asthma medication. Emphasized financial hardship due to illness.	described struggles getting medication even within the facility	Limited access to healthcare and medication might hinder treatment for SO${\;\;}_{2}$-related health issues.
*Environmental Damage*	Mentioned property damage (roofing sheets) due to air pollution, describes the impact on agriculture (burnt crops).	Mentioned damage to property (roofing sheets) due to air pollution.	Mentioned destroyed land and limited agricultural possibilities and impact on roofing sheets.	Environmental damage might affect exposure pathways and exacerbate health impacts.
*Coping Mechanisms*	Individual coping strategies, like home remedies and staying indoors, described feeling frustrated and helpless.	No specific mentions of individual coping strategies.	Shared experience of adapting routines and using home remedies, describes community frustration leading to occasional rioting in the past.	Individual coping strategies might not be sustainable, and limited community resources hinder effective response.
*Specific Concerns*	Mentioned nighttime releases of pollutants, described additional unidentified pollutants besides sulphur dioxide.	focused on acid production as a recent source of increased pollution. Further highlighted knowledge of specific emission days.	Mentioned nighttime releases of pollutants, focuses on silica dust exposure near the mine, shares experience with sulphur dioxide being released at night.	Nighttime releases could increase inhalation risks and suspected pollutants require investigation for potential health impacts.
*Main Demands*	Calls for improved healthcare access and medication availability, expresses the desire for relocation, compensation, and food support.	Places strongest emphasis on relocation as the primary solution, mentions the need for compensation and food support, desires financial resources for farming activities.	Calls for transparency and community involvement in monitoring, expresses a desire for relocation or improved living conditions.	Addressing demands could improve living conditions, reduce exposure, and enhance community engagement, which is crucial for managing SO_2_ health risks.

**Table 5:** Connecting community concerns with expert Insights: potential links to SO${\;\;}_{2}$ exposure.

Theme	Community Concerns (Focus Groups)	Clinic Officer Findings	Environmental Health Specialist Findings	Connection to Research Question
*Widespread Respiratory Problems*	Breathing difficulties, long-term health anxieties, short-term health effects, silica dust exposure (Group 3).	Positive trend in asthma cases after air quality monitoring installation, potential link between SO${\;\;}_{2}$ and respiratory ailments.	Detrimental impact of SO${\;\;}_{2}$ on respiratory health, citing emergency trays for asthma cases.	**Supports a potential link between reported health issues and SO${\;\;}_{2}$ inhalation. Clinic data aligns with concerns about long-term impacts.**
*Distrust in Monitoring Data*	Lack of trust in data accuracy, transparency demands, data manipulation concerns (Group 3).	Challenges in directly attributing illnesses to specific pollutants due to the unavailability of equipment for diagnosis.	Real-time monitoring of emissions has seen a reduction in both pollution and respiratory cases.	**Highlights need for more robust data collection, analysis, and transparency to address community concerns.**
*Limited Healthcare Access and Resources*	Inadequate healthcare access, medication burdens, healthcare facility challenges (Group 3).	Transition to digital systems for improved data management, limitations in advanced Equipment,	N/A (not directly discussed in this interview).	**Emphasizes the importance of addressing limited healthcare access and resources to improve treatment and preventive measures.**
*Environmental Damage Beyond Health*	Property damage, broader environmental consequences, damaged land, and limited agriculture (Group 3).	N/A (not directly discussed in this interview).	Detrimental effects of SO${\;\;}_{2}$ on both environment and health, including damage to vegetation, soil, and infrastructure.	**Confirms and expands upon community observations, underlining the urgency of addressing broader environmental impacts.**
*Specific Concerns and Demands*	Nighttime pollutant releases, unidentified pollutants, recent acid production increases (Group 2), silica dust and nighttime SO${\;\;}_{2}$ releases (Group 3).	Estimates 75% of treated respiratory cases potentially linked to environmental pollution, primarily SO${\;\;}_{2}$ emissions	N/A (not directly discussed in this interview).	**Provides valuable insights into specific community concerns and demands, guiding potential solutions and interventions.**

**Table 6:** Spearman correlations: SO_2_ vs. respiratory illnesses.

Respiratory Illnesses vs. ${\mathrm{SO}}_{2}$	Spearman Correlation Coefficient	*p*-Value	Correlation Interpretation	*p*-Value Significance
Asthma <1 year	−0.0001	0.9986	No correlation	Not Significant
Asthma (1–4) year	0.0237	0.7529	Weak positive correlation	Not Significant
Asthma >5 year	−0.5090	$3.4514\times 10^{-13}$	Strong negative correlation	Highly Significant
RI: non-P <1 year	−0.2111	0.0046	Moderate negative correlation	Significant
RI: non-P (1–4) year	−0.1312	0.0799	Weak negative correlation	Marginally Significant
RI: non-P >5 year	−0.3482	$1.7869\times 10^{-6}$	Moderate negative correlation	Highly Significant
RI: P <1 year	−0.0619	0.4103	Weak negative correlation	Not Significant
RI: P (1–4) year	0.0254	0.7360	Weak positive correlation	Not Significant
RI: P >5 year	−0.5734	$4.9335\times 10^{-17}$	Strong negative correlation	Highly Significant

## Data Availability

The corresponding author retains sole custody of all data obtained during this investigation. For privacy reasons, some information is not accessible to the public.

## Supplementary Materials

The following supporting information can be downloaded at: https://www.mdpi.com/article/10.3390/ijerph21070850/s1, Figure S1: Spatial Mapping; Table S1: Research Strategy; Datasets.
