# Supplementary material for: Unveiling the Nexus: Sulphur Dioxide Exposure, Proximity to Mining, and Respiratory Illnesses in Kankoyo: A Mixed-Methods Investigation
Source: Int J Environ Res Public Health. 2024 Jun 28;21(7):850. doi: 10.3390/ijerph21070850 (PMC11276504; doi:10.3390/ijerph21070850)
Supplement: Supplementary file 1 [file ijerph-21-00850-s001.zip › Table S1-Research Strategy.pdf]

Table S1: **Research Strategy for Investigating the Impact of SO_2 Exposure on Respiratory Health in Kankoyo, Mufulira, Zambia**

Phase	Objectives	Methodology	Outputs
**Phase 1: Contextualization and Planning**	Synthesize existing knowledge on SO_2 & respiratory health	Literature review (int'l & Zambian)	Gaps identified, research focus refined
	Assess regulatory landscape	Analyze Zambian environmental laws & monitoring data	Policy gaps/inconsistencies highlighted
	Gather preliminary insights	Initial community discussions & expert consultations	Qualitative insights to inform research design & questions
	Formulate research questions	Refine questions based on literature, preliminary insights, and policy analysis	Main research question and sub-research questions.
**Phase 2: Data Collection**	Understand community experiences & perceptions	Three focus groups with Kankoyo residents (n=24), expert interviews (n=2)	Qualitative data on community concerns & expert perspectives
	Map SO_2 hotspots	Spatial analysis using 2020 SO_2 data from 5 monitoring points, KDE heatmaps in QGIS	Spatial distribution of SO_2 hotspots, proximity to residents
	Analyze trends in SO_2 & respiratory illness over time	Retrospective analysis of health records and SO_2 data obtained from ZEMA and Copperbelt Provincial Health Office (2009-2023). Using Python programming.	Correlations, trends, patterns in SO_2 exposure & respiratory illnesses
**Phase 3: Data Integration and Discussion**	Synthesize findings to understand the SO_2-health connection	Triangulate data from all sources	Comprehensive understanding of SO_2-respiratory health link in Kankoyo
**Phase 4: Conclusion and Recommendations**	Draw conclusions & make recommendations	Based on integrated analysis, suggest policy changes, interventions, further research	Policy brief with recommendations for relevant stakeholders
	Share findings	Publications and presentations	Knowledge dissemination to empower community & inform decision-makers
