# Supplementary figures and images for: Unveiling the Nexus: Sulphur Dioxide Exposure, Proximity to Mining, and Respiratory Illnesses in Kankoyo: A Mixed-Methods Investigation
Source: Int J Environ Res Public Health. 2024 Jun 28;21(7):850. doi: 10.3390/ijerph21070850 (PMC11276504; doi:10.3390/ijerph21070850)

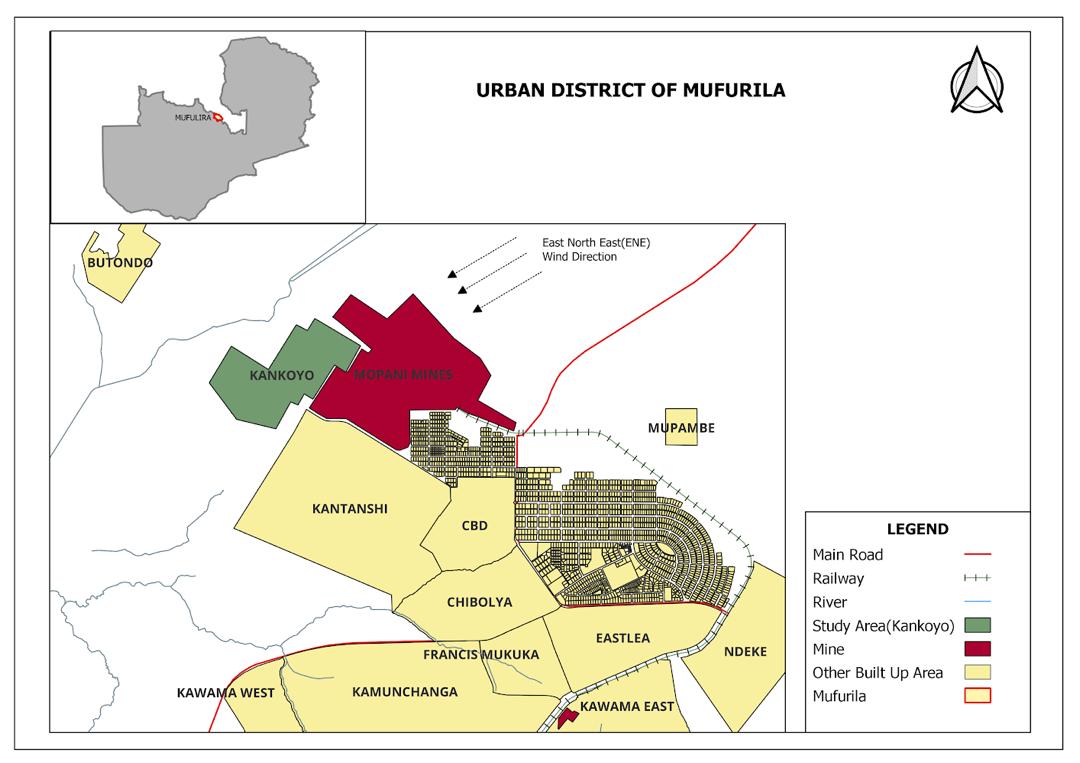

Supplement: Supplementary file 1 [file ijerph-21-00850-s001.zip › Figure S1-Spatial Mapping.jpeg]
